# Meta-analysis of the positive effect of aquatic fern (*Azolla pinnata*) intervention on growth performance metrics, blood chemistry, and carcass evaluation of broilers

**DOI:** 10.1007/s11250-026-05042-5

**Published:** 2026-04-17

**Authors:** C. A. Mbajiorgu, I. P. Ogbuewu

**Affiliations:** 1 Department of Agriculture and Animal Health, University of South Africa, Florida Science Campus, Private Bag X6, Florida, 1710 South Africa; 2 Department of Animal Science and Technology, Federal University of Technology, P.M.B. 1526, Owerri, Imo State Nigeria

**Keywords:** Broilers, Azolla pinnata, Feed conversion ratio, Average daily gain, Serum proteins, Serum lipids

## Abstract

This meta-analysis determined the impacts of aquatic fern (*Azolla pinnata*) on growth performance, serum chemistry, and carcass evaluation in broilers. Four electronic databases were searched for studies on the topic, and 1240 articles were identified and screened. Twenty-eight articles met the eligibility criteria and were used for the meta-analysis. Outcome measures were pooled using a random-effects model (REM) and expressed as mean difference (MD) at 95% confidence intervals (CI). The pooled results showed that *A. pinnata* (AP) significantly enhanced average daily gain (ADG) (*p* < 0.001) and feed conversion ratio (FCR) (*p* < 0.001) in broilers, taking into account significant heterogeneity. Furthermore, AP increased concentrations of total serum protein (*p* = 0.002), globulin (*p* < 0.001), and HDL (*p* < 0.001) in broilers. Results also revealed that AP significantly decreased serum total cholesterol (*p* < 0.001), triglycerides (*p* < 0.001), and LDL (*p* < 0.001) concentrations in broilers. Subgroup analysis revealed that Cobb broilers fed diets with 16–20% AP for 1–42 d had lower feed intake than the controls (*p* = 0.002), whereas Ross (*p* = 0.008) and Color synthetic (*p* < 0.001) had higher feed intake than the control. Ross fed diets with 0.1-5.0% and 6–10% AP for 1–21 d, 1–35 d, and 22–42 d, which had superior FCR and ADG compared to the control. Meta-regression results showed that broiler strains as a moderator explained 19–34% of heterogeneity in feed intake, FCR and ADG in broilers fed diets containing AP. In conclusion, the present study demonstrated that AP can be added to broiler feed at levels up to 10% to enhance growth performance and health. This study provides important information for policy advancements and sustainable utilization of AP as a feed ingredient in the modern broiler industry, promoting efficient and environmentally friendly poultry production.

**Supplementary Information:**

The online version contains supplementary material available at 10.1007/s11250-026-05042-5.

## Introduction

Broiler farming plays a pivotal role in global food and nutrition security, providing a rich source of high-quality protein to meet the growing demand for poultry meat (Vlaicu et al. 2024). However, broiler meat is considered a luxury by resource-poor households in developing nations due to the high cost of chicken products, mainly driven by the high cost of feed, which accounts for up to 70% of total production expenses (Alem 2024). This has led poultry nutritionists to explore sustainable alternatives to conventional protein sources like soybean meal. The use of aquatic ferns in recent times as sustainable protein sources in broiler nutrition is on the rise due to their nutritional and pharmacological benefits (El-Bahr et al. 2020; Mokhtar et al. 2020; Eid Abdel-Moneim et al. 2022). *Azolla pinnata* (AP), one such aquatic ferns belong to the family Salviniaceae and is native to Africa, Asia, and parts of Australia. It is a highly productive plant with rich in minerals, vitamins, crude protein (20–30%), and amino acids (Mokhtar et al. 2020; Riaz et al. 2022). Azolla pinnata has a higher essential amino acid (EAA) index than fish meal, soybean meal, cottonseed meal, and canola seed meal (Moyo and Rapatsa-Malatji 2023). However, its use in poultry nutrition is limited by the presence of anti-nutritional factors (ANFs), including tannins and phytates, which inhibit digestibility and nutrient absorption at high inclusion levels in broiler diets (Alem 2024). Nevertheless, research has shown that Azolla contains numerous phytochemicals, including beta-carotenes, phenols, and flavonoids (Bouattou et al. 2024). These beneficial phytochemicals contribute to their various medicinal properties, including antioxidants, cardioprotective, antifungal, antimicrobial, anti-inflammatory, and immuno-stimulatory (Riaz et al. 2022; Hafeez et al. 2024).

Studies on the impacts of AP on broiler productivity and health have yielded inconsistent outcomes (Samad et al. 2020; Riaz et al. 2022). Some studies revealed improved growth performance, nutrient digestibility, absorptive capacity of the intestines, and modulated gut microbiota composition in broilers (Samad et al. 2020; Abdelatty et al. 2021; Liu et al. 2022), while others found negative or no effects (Abdelatty et al. 2020; Ali et al. 2022; Alem 2024). These variable outcomes hinder the use of this valuable information in evidence-based decision-making in the poultry industry.

The variable outcomes may be linked to modifiers like AP composition, processing methods, quantity of AP added to the feed, and broiler characteristics. Attempts to quantify the amount of heterogeneity explained by these modifiers in broilers fed diets containing AP using the traditional review methodology did not yield the desired results, as traditional review lacks the methodical rigor required to quantify the observed variability (Riaz et al. 2022; Alem 2024). The use of meta-analysis, an advanced statistical method that combines findings from several independent studies on the same topic to reconcile inconsistent findings and increase statistical power in the animal science discipline, has been reported (Herath et al. 2023; Ogbuewu et al. 2024). Given that the goal of the modern poultry industry is to search for novel protein feed resources with the potential to relieve pressure on traditional plant protein sources in broiler feed. Therefore, the objective of this meta-analysis was to determine the positive effects of *Azolla pinnata* on growth performance, blood chemistry, and carcass characteristics of broilers. It hypothesised that dietary *Azolla pinnata* intervention has positive effects on growth performance metrics and blood chemistry of broilers.

## Materials and methods

### Search strategy

The PRISMA (Preferred Reporting Items for Systematic reviews and Meta-Analyses) guidelines were used for the study. PubMed, Scopus, Web of Science, and Google Scholar were searched to identify relevant studies on the effect of dietary AP intervention on broiler performance from 1st to 10th June 2025. The choice of four electronic databases was to minimize biased results due to the scope or journal coverage of one database (DeSimone et al. 2020; Martín-Martín et al. 2021). The search keywords were “broilers”, “broiler chickens”, “Azolla”, “Azolla plant”, *“Azolla pinnata*”, “growth performance”, “organ weight”, “carcass”, “serum biochemistry”, “blood chemistry”, “clinical chemistry”, “liver function enzymes”, and “serum lipid”, and “blood lipids”. The search queries were Boolean operators (AND/OR) and wildcards (* and? ).

### Eligibility criteria

**Fig. 1:**
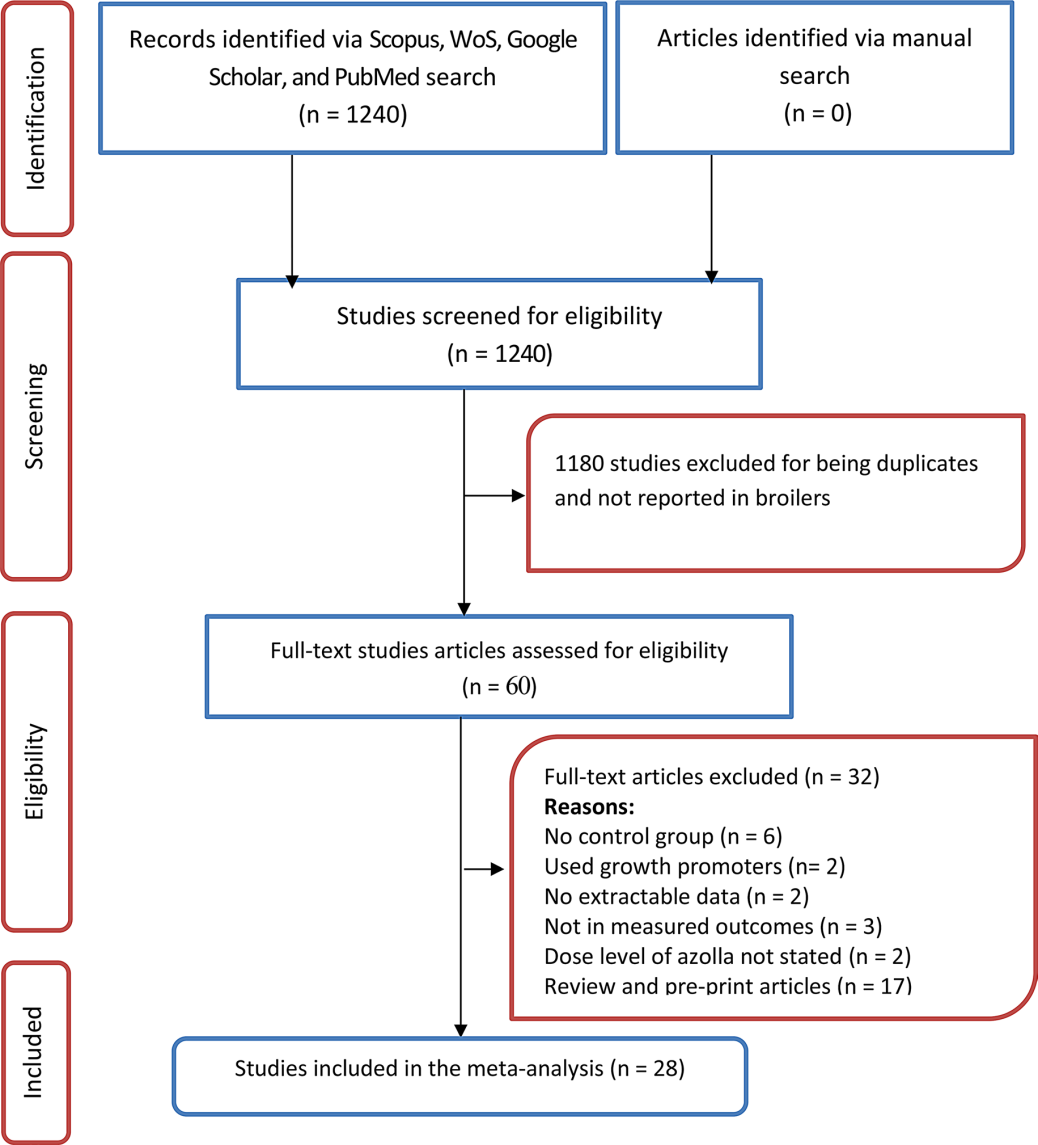
PRISMA selection flow chart of the included studies

Studies were identified using the PICO (P: population, I: intervention; C: comparators, and O: outcomes) template as shown in Table 1. Inclusion criteria were (1) studies that assessed the effect of a diet with and without AP on at least one of the measured outcomes in broilers: (2) studies that fed diets that were free from antibiotics or other growth-promoting agents, and (3) studies that reported the means of the measured outcomes for the control and experimental groups. Exclusion criteria included: (1) Research not performed in broilers, (2) trials not on measured outcomes of interest, (3) studies without control group, (4) reviews articles, (5) studies with no extractable data, (6) duplicate studies and preprints, and (7) studies that did not state the amount of azolla added to the diet. The PRISMA flow chart as displayed in Fig. 1 shows that 1240 studies were identified from the search done on the four bibliographic databases, of which 28 met the eligibility criteria.

**Table 1:** PICO format for the study

PICO	Description
Population (P):	Broilers
Intervention (I):	Diet with *Azolla pinnata*
Comparators (C):	Diet without *Azolla pinnata*
Outcomes (O):	Growth performance: Average daily gain, feed intake, feed conversion ratio)
	Blood metabolites: Total protein, albumin, globulin, urea, creatinine, triglycerides, total cholesterol, high density-lipoprotein, low density-lipoprotein, aspartate transferase, and alanine transferase.
	Carcass traits: Dressing percentage, breast, thigh, drumstick, and abdominal fat.
	Organ weight: Liver, heart, gizzard, and spleen.

### Data extraction and statistical analysis

Data on study characteristics (surname of the first author, publication year, and study location) and measured outcomes were extracted from each eligible study. Data was also extracted on the following covariates: broiler strains (Arbor Acres, Color Synthetic, Cobb, Ross, and Hubbard), inclusion level (0.1-5, 6–10, 11–15, 16–20, and 21–25, and 26–30%), and production phases (1–21, 1–35, 1–42, and 22–42 d) considered to influence the outcomes of the pooled results were extracted. Data presented in graphs, bars, and pie charts were extracted using WebplotDigitizer Version 4.5 (Rohatgi 2021). A database of 28 candidate studies that met the eligibility criteria was built, as shown in Table S1.

### Effect size

Mean difference (MD) was used to calculate the effect of diets containing AP on broiler productivity and health. MD provides a clear advantage over other metrics, as it represents the actual difference in means on the original measurement scale, making it intuitively understandable and readily interpretable. A random-effect model (REM) was chosen over a fixed-effect model to account for expected variability between studies (McKenzie and Veroniki 2024). This model accounts for variations between studies, providing a more accurate estimate. The potential sources of heterogeneity considered in this study were the production phase, AP dosage, and broiler strains.

## Moderator analysis and publication bias

Heterogeneity was assessed using the Q-statistic and I^2^-statistics (low: 25%, moderate: 25–75%, and high: >75%) (Nyaga and Arbyn 2024). The I^2^-statistic represents the percentage of difference in a meta-analysis that is due to heterogeneity between studies, rather than sampling error. Sensitivity analysis was conducted via the leave-one-out method to evaluate the influence of individual studies on pooled estimates. Meta-regression was performed to explore the effect of individual covariates on the observed heterogeneity. Meta-regression was conducted only on measured outcomes with a minimum of 10 studies to increase statistical power (Hernandez-García et al. 2024). Subgroup analysis was stratified for covariates with 3 comparisons in each stratum or more (Ogbuewu et al. 2024). Publication bias was assessed visually via funnel plots and quantitatively using Egger’s regression test, with *p* < 0.05 indicating bias.

### Statistical analysis

Analyses were done using OpenMEE software (Wallace et al. 2016) and R software (version 4.3.0) at a significance level of *P* < 0.05. Pooled results were displayed as MD with 95% CI using a random effect model.

## Results

### Search results and study characteristics

**Fig. 2:**
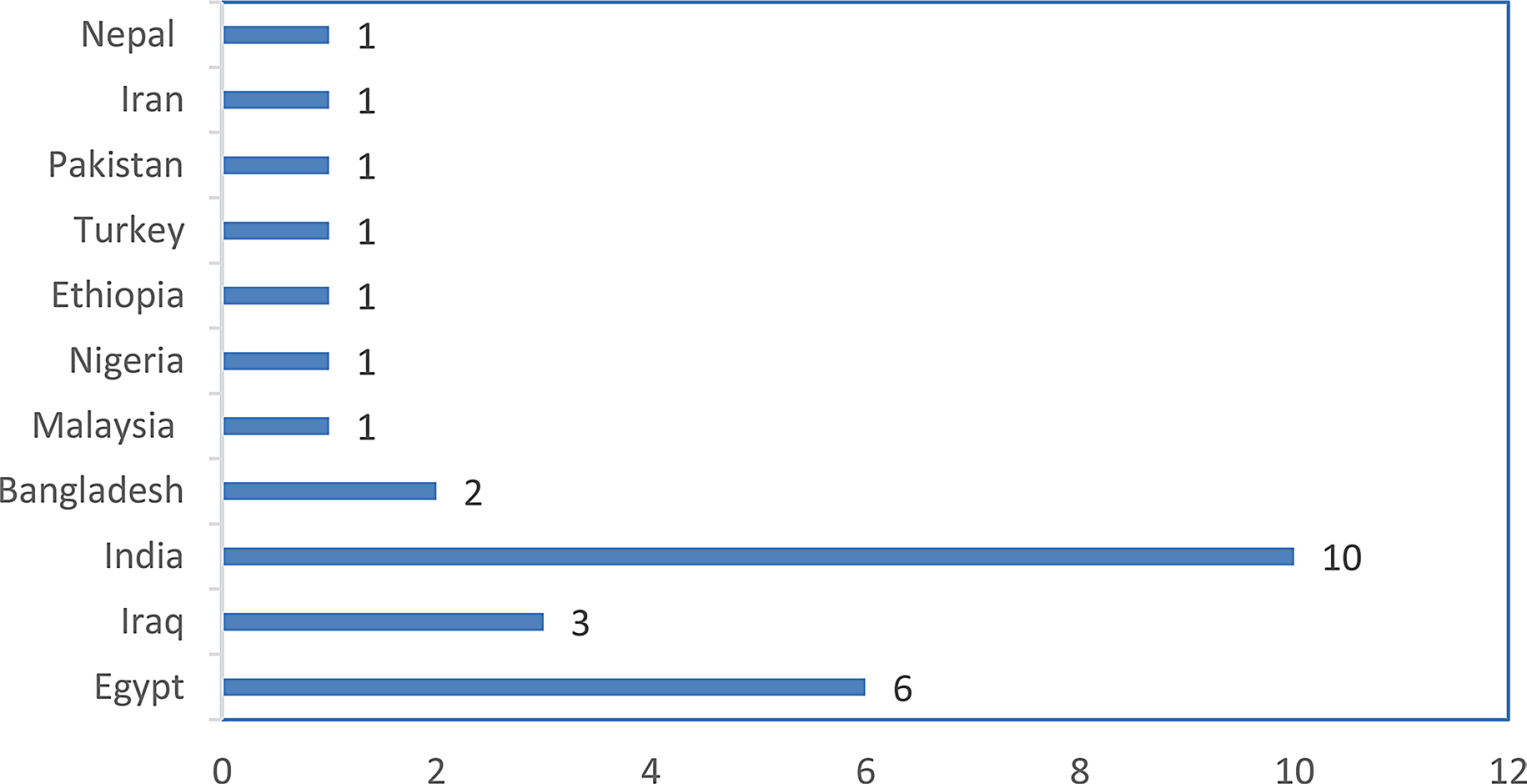
Study continent

**Fig. 3:**
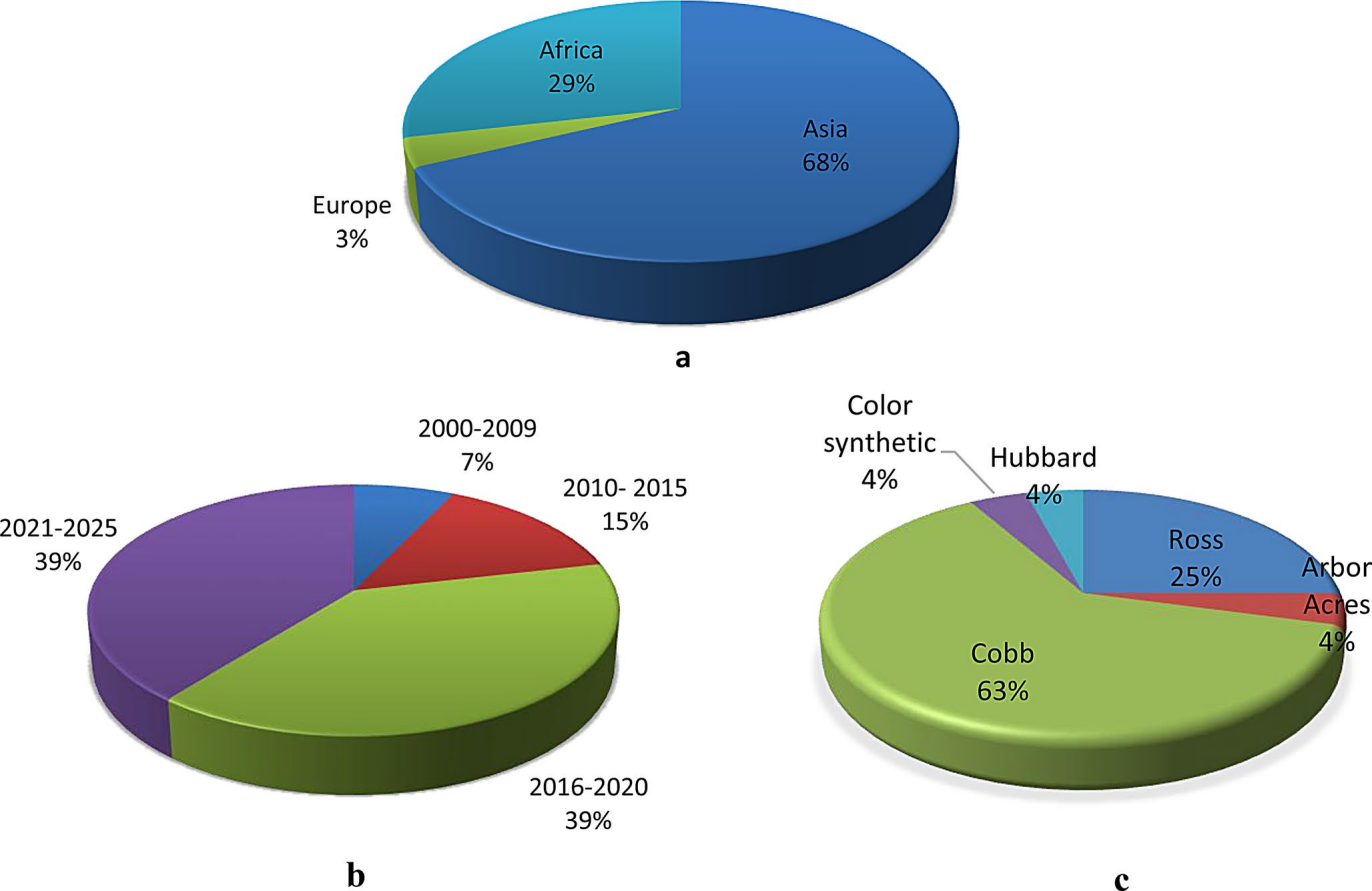
Distribution matrix of (**a**) study continent, (**b**) publication year and (**c**) broiler strains used for the study

The search produced 1240 studies on the topic, and 28 met the eligibility criteria (Fig. 1). Supplementary Table S1 summarises the characteristics of the 28 eligible studies. Of the 28 studies used for the meta-analysis, 10, 6, 3, and 2 were conducted in India, Egypt, Iraq, and Bangladesh, respectively (Fig. 2). Most of the trials were performed in Asia (68%) followed by Africa (29%), as illustrated in Fig. 3a. The majority of the research was done in 2016–2020 and 2021–2025 (Fig. 3b). Cobb (63%) and Ross (25%) were the most studied strains (Fig. 3c).

### Growth performance and analysis of covariates

Pooled estimation as shown in Table 2 revealed that AP improved FCR (*p* < 0.001) and ADG (*p* < 0.001) in broilers compared to controls. In contrast, broilers fed diets containing AP had a similar feed intake to the control (*p* = 0.588). There is also evidence of large heterogeneity across the 28 studies used for the meta-analysis (*I*^*2*^-statistic = 91–93%; *p* < 0.001; Table 2). There is also evidence of publication bias across studies that assessed the impact of AP intervention on FCR and ADG in broilers, as presented in Table 2 and Supplementary Figure S1. Fail-safe number (Nfs) found that the observed p-value of < 0.0001 was less than the target significance level of 0.05, and this also confirmed the presence of publication bias for studies that evaluated the effect of AP on FCR and ADG in broilers. However, Nfs for the database were 949 (ADG) and 1216 (FCR), which were two times higher than the values of 525 (5*103 + 10) and 550 (5*108 + 10) needed to declare the mean effect size significant despite the possibility of publication bias.

**Table 2:** Pooled effect of *Azolla pinnata* on growth dynamics of broilers

Outcomes	Model Results			Heterogeneity					ET
	MD	95% CI	*p*-value	T^2^	Q	df	*p*-value	I^2^	
FI	-0.04 g/bird	-0.18, 0.10	0.588	0.47	1145.60	102	< 0.001	91	0.152
FCR	-0.70 g/bird	-0.46, -0.14	< 0.001	0.68	1571.32	107	< 0.001	93	0.043
ADG	0.88 g/bird	0.12, 0.45	< 0.001	0.67	1493.01	102	< 0.001	93	0.032

*MD* mean difference; *FCR* feed conversion ratio; *ADG* average daily gain; *ET* egger test; *DF* degree of freedom; *T*^*2*^ tau-squared; *P* probability

Subgroup analysis as presented in Table 3 indicates that Cobb broilers fed diets containing 16–20% AP for 1–42 d had significantly lower feed intake than the controls. On the other hand, Ross strain (*p* = 0.008) and Color Synthetic strain (*p* < 0.001) had significantly higher feed intake than the controls. Table 4 revealed that Ross, Arbor Acres, Color Synthetic, and Hubbard strains fed diets having 0.1–5% and 6–10% AP for 1–21 d, 1–35 d, and 22–42 d had superior FCR than the controls. Table 5 found that Ross, Color Synthetic, and Hubbard offered AP at 0.1-5%, 6–10%, and 16–20% for 1–21 d, 1–35 d, and 22–42 d had superior ADG values than the controls. Meta-regression showing the relationship between covariates and growth performance metrics in broilers is presented in Table 6. Broiler strains and dosage were significantly related to the feed intake (*p* < 0.001) and explained 13–30% of the observed variability. The production phase was not significantly associated with feed intake (*p* = 0.059) and FCR (*p* = 0.661). In contrast, broiler strains were associated with FCR (*p* < 0.001), and results show little effect of strain as a covariate (R^2^ = 19%). Meta-regression found significant associations between ADG and covariates: broiler strains (*p* < 0.001; R^2^ = 34%), dosage (*p* = 0.003; R^2^ = 11%), and production phase (*p* = 0.002; R^2^ = 11%) in broilers.

**Table 3:** Impact of covariates on feed intake of broilers offered *Azolla pinnata*-based diets

Covariates	Subgroups	*n*	MD	95% CI		SE	*p*-value
				LL	UL		
PP (day)	1–21	25	0.15	-0.08	0.39	0.12	0.204
	1–35	24	0.00	-0.19	0.36	0.14	0.546
	1–42	44	-0.31	-0.56	-0.06	0.13	0.014
	22–42	14	0.27	-0.09	0.62	0.18	0.141
Dosage (%)	0.1-5	44	-0.01	-0.14	0.13	0.07	0.896
	6–10	33	0.15	-0.15	0.46	0.16	0.323
	11–15	21	0.09	-0.31	0.48	0.20	0.669
	16–20	7	-0.89	-1.59	-0.20	0.36	0.012
Broiler strains	Ross	13	0.38	0.10	0.66	0.14	0.008
	Cobb	52	-0.30	-0.49	-0.11	0.10	0.002
	Arbor Acres	12	0.00	-0.47	0.47	0.24	0.989
	Color synthetic	6	1.79	1.34	2.24	0.23	< 0.001
	Hubbard	3	0.02	-0.51	0.55	0.27	0.951

MD mean difference; n datasets; LL lower limit; UL upper limit; PP production phase; MD mean difference; CI confidence interval; SE standard error; p probability

**Table 4:** Impact of covariates on FCR of broilers fed *Azolla pinnata*-based diets

Covariates	Subgroups	*n*	MD	95% CI		SE	*p*-value
				LL	UL		
PP (day)	1–21	24	-0.34	-0.70	-0.52	0.18	0.044
	1–35	27	-0.38	-0.65	-0.10	0.14	0.007
	1–42	40	-0.15	-0.46	0.16	0.16	0.330
	22–42	17	-0.44	-0.72	-0.17	0.14	0.001
Dosage (%)	0.1-5	47	-0.33	-0.52	-0.14	0.10	< 0.001
	6–10	37	-0.30	-0.57	-0.02	0.14	0.034
	11–15	16	-0.17	-0.87	0.53	0.36	0.628
	16–20	6	0.02	-0.68	0.73	0.36	0.946
Broiler strains	Ross	22	-0.37	-0.61	-0.13	0.12	0.003
	Cobb	48	-0.08	-0.34	0.18	0.13	0.565
	Arbor Acres	12	-0.78	-1.29	-0.26	0.26	0.003
	Color synthetic	6	-1.55	-2.19	-0.91	0.33	< 0.001
	Hubbard	3	1.25	0.61	1.88	0.33	< 0.001

MD mean difference; FCR feed conversion ratio; n datasets; PP production phase; LL lower limit; UL upper limit; CI confidence interval; SE standard error; p probability

**Table 5:** Effect of covariates on ADG of broilers fed *Azolla pinnata*-based diets

Covariates	Subgroups	*n*	MD	95% CI		SE	*p*-value
				LL	UL		
PP (day)	1–21	24	0.44	0.10	0.79	0.18	0.012
	1–35	22	0.47	0.09	0.86	0.20	0.016
	1–42	38	-0.17	-0.41	0.08	0.12	0.183
	22–42	19	0.80	0.37	1.20	0.21	< 0.001
Dosage (%)	0.1-5	45	0.37	0.17	0.57	0.10	< 0.001
	6–10	34	0.51	0.22	0.80	0.15	< 0.001
	11–15	16	0.16	-0.44	0.75	0.30	0.602
	16–20	6	-0.98	-1.59	-0.38	0.31	0.001
Broiler strains	Cobb	46	-0.03	-0.24	0.19	0.11	0.812
	Ross	19	0.62	0.33	0.90	0.15	< 0.001
	Arbor Acres	12	0.75	-0.07	1.59	0.43	0.079
	Color synthetic	6	2.20	1.94	2.47	0.13	< 0.001
	Hubbard	3	-1.22	-2.36	-0.07	0.58	0.037

MD mean difference; ADG average daily gain; n datasets; PP production phase; LL lower limit; UL upper limit; MD mean difference; CI confidence interval; SE standard error; p probability

**Table 6:** Relationship between outcomes and covariates

Outcomes	Covariates	β	Q_C_	df	*p*-value	*R*^2^ (%)
Feed intake	Broiler strains	0.38	36.3	4	< 0.001	30
	Production phase	-0.31	7.41	3	0.059	4
	Dosage	-0.02	19.5	4	< 0.001	13
FCR	Broiler strains	-0.391	23.8	4	< 0.001	19
	Production phase	-0.15	1.59	3	0.661	0
	Dosage	-0.34	3.54	4	0.472	0
ADG	Broiler strains	0.635	43	3	< 0.001	34
	Production phase	-0.174	14.8	3	0.002	11
	Dosage	0.384	16.1	4	0.003	11

FCR feed conversion ratio; ADG average daily gain; Q_M_ coefficient of covariates; df degree of freedom; β meta-regression coefficient; p probability; R^2^ the amount of heterogeneity accounted for

### Carcass and organ weight characteristics

Table 7 presents the effect of AP intervention on organ weights and carcass traits of broilers. Dietary AP intervention improved heart weight compared with the control (*p* < 0.001). In contrast, the weights of gizzard (*p* = 0.646), liver (*p* = 0.393), and spleen (*p* = 0.681) in broilers fed diets containing AP were not statistically different from controls. Likewise, there were no significant differences in dressing percentage (*p* = 0.139), breast (*p* = 0.388), thigh (*p* = 0.298), drumstick (*p* = 0.152), and abdominal fat (*p* = 0.669) in the two groups. There was no evidence of publication bias as shown by the Egger regression test. There was significant heterogeneity across the articles used for the meta-analysis (*I*^*2*^-statistic = 52–97%).

**Table 7:** Pooled effect of *Azolla pinnata* on organ weight and carcass traits of broilers

Outcomes (g)	Model Results			Heterogeneity				
	MD	95% CI	*p*-val	T^2^	Q	df	*p*-value	I^2^
Gizzard	0.07	-0.24, 0.39	0.646	0.36	141.13	15	< 0.001	89
Heart	0.75	0.40, 1.11	< 0.001	0.24	43.45	8	< 0.001	82
Liver	0.09	-0.12, 0.29	0.393	0.12	59.77	15	< 0.001	75
Spleen	0.17	-0.65, 0.99	0.681	2.01	347.25	11	< 0.001	97
DP	0.13	-0.04, 0.31	0.139	0.07	38.27	14	< 0.001	63
Breast	-0.08	-0.26, 0.10	0.388	0.06	28.50	11	0.003	61
Drumstick	0.23	-0.09, 0.55	0.152	0.16	33.42	7	< 0.001	79
Thigh	0.08	-0.07, 0.23	0.298	0.04	24.82	12	0.016	52
Abdominal fat	0.02	-0.08, 0.13	0.669	0.02	92.65	7	< 0.001	92

MD mean difference; DP dressing percentage; ET egger test; dF degree of freedom; I^2^ Inconsistency index; T^2^ tau-squared; Q Cochran statistic; p probability

### Serum biochemical characteristics

Table 8 presents the results of the effect of dietary AP intervention on the serum biochemical parameters of broilers. Dietary AP intervention increased total protein (*p* = 0.002) and globulin (*p* < 0.001) in broilers compared to controls without evidence of publication bias. On the other hand, there were no significant differences in concentrations of serum albumin (*p* = 0.465), urea (*p* = 0.561), creatinine (*p* = 0.107), AST (*p* = 0.467), and ALT (*p* = 0.761) between the two groups. The results show a significant decline in serum total cholesterol levels (*p* < 0.001), triglycerides (*p* < 0.001), and LDL (*p* < 0.001) in comparison with controls. In contrast, dietary AP intervention improved serum HDL levels compared with the control (*p* < 0.001). There was significant heterogeneity across the articles used for the meta-analysis (*I*^*2*^-statistic = 93–98%).

**Table 8:** Pooled estimation of *Azolla pinnata* on blood chemistry parameters of broilers

Outcomes	Model Results			Heterogeneity				
	MD	95% CI	*p*-value	T^2^	Q	df	*p*-value	I^2^
Total protein (g/dl)	0.69	0.26, 1.13	0.002	0.92	261.50	19	< 0.001	93
Albumin (g/dl)	0.25	-0.42, 0.92	0.465	1.77	362.41	15	< 0.001	96
Globulin (g/dl)	1.19	0.51, 1.87	< 0.001	1.81	354.47	15	< 0.001	96
Urea (mg/dl)	-0.30	-1.32, 0.72	0.561	4.46	808.47	16	< 0.001	98
Creatinine (mg/dl)	0.71	-0.15, 1.56	0.107	2.40	431.39	12	< 0.001	97
Triglycerides (mg/dl)	-0.14	-0.83, -0.55	< 0.001	2.39	582.85	19	< 0.001	97
TC (mg/dl)	-0.50	-1.45, -0.45	< 0.001	4.56	906.10	19	< 0.001	98
HDL (mg/dl)	0.69	0.12, 1.49	< 0.001	2.61	464.63	15	< 0.001	97
LDL (mg/dl)	-0.93	-2.46, -0.61	< 0.001	7.83	661.92	12	< 0.001	98
ALT (mg/dl)	0.17	-0.95, 1.30	0.761	5.45	911.42	16	< 0.001	98
AST (mg/dl)	-0.34	-1.24, 0.57	0.467	3.53	689.23	16	< 0.001	98

MD mean difference; TC total cholesterol; HDL high density lipoprotein; LDL low density lipoprotein; AST aspartate aminotransferase; ALT alanine aminotransferase; ET egger test; dF degree of freedom; I^2^ Inconsistency index; T^2^ tau-squared; Q Cochran statistic; P probability

## Discussion

The pooled results revealed that AP improved FCR and ADG in broilers without affecting feed intake. Azolla pinnata carry out its functions including improving FCR and ADG through different mechanisms which in broilers include improved nutrient digestibility, modulation of gut health toward a more efficient absorptive state, and enhanced metabolic efficiency due to rich bioactive compounds, high protein content, and balance micronutrient profiles (Abdelatty et al. 2021; Mokhtar et al. 2020; Riaz et al. 2022; Moyo and Rapatsa-Malatji 2023; Khan et al. 2024). Additionally, AP possesses a favorable amino acid profile, including essential amino acids such as lysine, which are critical for protein synthesis and muscle deposition. This observation indicates that AP acts as a functional feed ingredient that enhances nutrient utilisation rather than simply increasing feed intake. In contrast to the current findings, Abdelatty et al. (2020) reported that inclusion of up to 10% AP in broiler diets had no significant effect on FCR, and ADG. The reason for this discrepancy is not clear. However, it may be attributed to the relatively small number of broilers used in their study, which likely limited the statistical power to detect the true effects of AP on broiler performance (Brydges 2019).

The subgroup fed 16–20% AP for 1–42 days had decreased feed intake compared to the control, suggesting that feeding high levels of AP for 42 days may negatively impact feed intake in broilers. This finding aligns with Yassar et al. (2025), who observed a significant decrease in feed intake among Cobb 500 broilers fed 20% and 30% AP for 42 days. The reduced performance could be due to anti-nutritional factors in AP, such as tannins and phytate (Alem 2024). These compounds may compromise broiler performance when AP is fed at higher levels (Abdelatty et al. 2021; Yassar et al. 2025). Further research is needed to determine the inclusion levels of AP that optimize feed intake in broilers, as such data is limited in the literature. In the present study, the Ross strain that received diets containing AP consumed more feed than the control, whereas the Cobb strain showed the opposite trend. This supports the meta-regression results, which identified broiler strains as a significant predictor of feed intake in broilers fed diets containing AP. Notably, AP had a large positive effect on feed intake in the Color Synthetic strain. However, this finding should be interpreted cautiously due to the small sample size.

The subgroup analysis showed that broilers fed diets containing AP for 1–21, 1–35 and 22–42 d had improved FCR. Notably, the improved FCR during the finisher phase (22–42 days) suggests that adult broilers can effectively utilize nutrients and bioactive compounds in diets containing AP, resulting in enhanced ADG. Broilers fed AP at 0.1-5.0% and 6–10% inclusion levels had significantly reduced FCR, indicating effective utilization of these diets (Abdelatty et al. 2021). Results indicate that strain is a significant predictor of FCR in broilers offered AP diets, accounting for 19% sources of heterogeneity. Consistent with this finding, the subgroup results demonstrated that Ross, Arbor Acres, and Color synthetic strains had better FCR compared to the Hubbard strain. These findings align with Durosaro et al. (2023), who reported that genotype affects FCR in chickens.

The significant increase in ADG in broilers fed AP at 0.1-5.0% and 6–10% for 1–21 d, 1–35 d, and 22–42 d compared with the controls could be related to AP’s high protein and excellent amino acid profile (Leterme et al. 2009; Moyo and Rapatsa-Malatji 2023). The improved ADG in broilers fed AP at 0.1-5.0% and 6–10% for 1–21, 1–35, and 22–42 d indicates that low inclusion levels of AP support ADG in broilers. This finding corroborates the earlier results of Abdelatty et al. (2021) in broilers. Broilers fed 16–20% AP had poor ADG, implying a reduced ability to support muscle growth. This may be due to high levels of anti-nutritional compounds (e.g., fiber, phytates, oxalates, and tannins) in the AP diet, exceeding the threshold that broilers can utilize (Abdelatty et al. 2021). These findings showed that strain influenced ADG in broilers, with Ross, Arbor Acres, and Color Synthetic showing lower ability to convert diets containing AP to muscles. In contrast, the Hubbard strain exhibited increased ADG, suggesting a high ability of AP to support muscle growth. However, this finding should be interpreted with caution due to the small sample size. This result supports the view of Durosaro et al. (2023) that chicken genetics influence FCR and ADG.

*Azolla pinnata* contains antioxidant compounds like phenolic acids and flavonoids, which help mitigate oxidative stress by scavenging free radicals (Bouattou et al. 2024). This study found that broilers fed diets containing AP had increased heart weights, which could be attributed to its cardioprotective effects (Rahman et al. 2023; Bouattou et al. 2024). Serum metabolites are used to evaluate the nutritional quality of novel feed resources (Ogbuewu and Mbajiorgu 2024; Abd El-Kareem et al. 2025). The higher total serum protein in AP-fed broilers indicates improved protein metabolism and immune functions (Riaz et al. 2022). Furthermore, the increased serum globulin levels in AP-fed broilers support the immunostimulatory properties of AP (Hafeez et al. 2024), suggesting its potential benefits in poultry nutrition.

Clinical data strongly support that nutrition affects serum cholesterol levels in animals (Abd El-Kareem et al. 2025). The current meta-analysis revealed that AP altered lipid profiles in broilers by decreasing total cholesterol, triglycerides, and LDL concentrations, while increasing HDL levels. Although the exact mechanism by which AP enhances serum lipid profiles in broilers is not clear. However, Azolla’s phytochemical components (i.e., flavonoids, tannins, and phenolic compounds) may regulate lipid metabolism by influencing gene expression related to lipid metabolism, enhancing bile acid excretion, and suppressing the activity of HMG-CoA reductase, a key enzyme in cholesterol synthesis (Sun et al. 2018; Hamouda et al. 2024).

Heterogeneity in studies reflects underlying differences in broiler genetics, differences in study design and analytical methods, and statistical variation around responses (Lean et al. 2009). There was evidence of large heterogeneity across studies used for the meta-analysis, which could be attributed to factors such as nutritional quality of the Azolla, processing methods, quantity added to the diet, diet formulation, and environmental conditions, broiler strain, age of broilers, and several others. Results revealed that restricted subgroup and sensitivity analyses did not detect the sources of heterogeneity. Nonetheless, meta-regression analysis revealed that the analyzed covariates (production phase, dosage, and broiler strain) explained some of the sources of heterogeneity in the pooled results. The unaccounted heterogeneity could be ascribed to several factors, including variations in environmental temperature and light intensity during Azolla cultivation, differences in nutritional quality of the Azolla, drying method (sun-drying versus oven-drying), presence of antinutritional factors, processing methods, inclusion levels in the diet, and overall diet composition, and litter quality. These factors were not evaluated in the present meta-analysis due to limited studies in this area. Publication bias, which arises when the findings of a study influence the decision to publish the study or not by the journal editor, is a serious issue in meta-analysis since it undermines the credibility of the pooled outcomes (Ogbuewu et al. 2024). One possible explanation for the observed publication bias in this meta-analysis is that studies reporting improved broiler performance with dietary Azolla may be more attractive to journal editors and thus more likely to be published. Additionally, there is a strong drive to find cost-effective alternatives to expensive conventional protein sources such as soybean meal and fishmeal. This motivation may create a bias toward publishing studies that support positive effects of such alternatives, thereby supporting the desired cost-reduction narrative. However, publication bias was not an issue in this study because it would take a large number of unpublished non-significant studies to reduce the statistical significance of diets containing AP on ADG and FCR to non-significance (Peters and Verhagen 2024).

### Conclusion and future research direction

This study demonstrated that 6–10% AP can be included in broiler diet for 1–21 d, 1–35 d, and 22–42 d to improved FCR and ADG in broilers without affecting feed intake, suggesting that replacing a certain percentage of conventional protein feed resource with 6–10% AP, improves FCR, ADG and reduce feed cost. The results indicate that AP enhanced heart weight and lipid profiles in broilers. The subgroup results showed that the Cobb fed 16–20% AP for 1–42 days had lower feed intake than the controls, while the reverse was the case for Ross and Color Synthetic. In addition, Ross fed 0.1-5% and 6–10% AP for 1–21 d, 1–35 d, and 22–42 d had improved FCR and ADG. Meta-regression showed that broiler strain was a significant predictor of feed intake, FCR, and ADG in broilers fed diets containing AP and explained 19–34% of the sources of heterogeneity. However, additional research is needed to identify other factors responsible for the unexplained variability. Evidently, AP is readily available and easily sourced in many tropical and subtropical localities, as it grows rapidly in ponds or rice fields all year round with minimal inputs. Studies highlight its high production capacity (up to 750 tonnes/acre) and very low production cost. However, there is a research gap in the economics of the production of broilers fed AP, as well as the exact inclusion levels of AP that optimize broiler performance. Thus, future research should focus on these areas. Future research and extension efforts should focus on disseminating effective cultivation techniques and developing region-specific feeding guidelines to promote wider adoption of AP in broiler production. It is recommended that biotechnological techniques be employed to improve the nutritional quality of AP so that it can be included at higher levels in the broiler diet. This study provides important information for policy advancements and sustainable utilization of AP as a nutrient-rich protein feedstuff in broiler production.

## Supplementary Information

Below is the link to the electronic supplementary material.

Supplementary Material 1

## Data Availability

Data will be made available on reasonable request.
