# Supplementary material for: Meta-analysis of the positive effect of aquatic fern (Azolla pinnata) intervention on growth performance metrics, blood chemistry, and carcass evaluation of broilers
Source: Trop Anim Health Prod. 2026 Apr 17;58(4):237. doi: 10.1007/s11250-026-05042-5 (PMC13086671; doi:10.1007/s11250-026-05042-5)
Supplement: Supplementary file 1 — Supplementary Material 1 [file 11250_2026_5042_MOESM1_ESM.docx]

**Supplementary Table S1:** Characteristics of studies included in the meta-analysis

| s/n | Authors | Location | Continent | Covariates | | | Outcomes |
| --- | --- | --- | --- | --- | --- | --- | --- |
|  |  |  |  | Broiler strains | PP (day) | Dosage (%) |  |
| 1 | Abdelatty et al. (2020) | Egypt | Africa | Ross | 1.0-42 | 0, 5-10 | GP, BC, CT |
| 2 | Abdelatty et al. (2021) | Egypt | Africa | Ross | 1.0-35 | 0, 5-10 | GP, OW |
| 3 | Hamed and Al- Husseiny (2023) | Iraq | Asia | Ross | 1.0-42 | 0, 4.5-13.5 | GP |
| 4 | Arram et al. (2023) | Egypt | Africa | AA | 1.0-35 | 0, 5-20 | GP, BC |
| 5 | Dhumal et al. (2009) | India | Asia | Cobb | 1.0-21 | 0, 2.5-5 | GP, OW |
| 6 | Abd El-Kareem et al. (2025) | Egypt | Africa | Ross | 1.0-21 | 0, 3-9 | GP, BC |
| 7 | Islam and Nishibori (2017) | Bangladesh | Asia | Cobb | 1.0-35 | 0, 5-7 | GP |
| 8 | Kamel and Hamed (2021) | Egypt | Africa | Cobb | 1.0-42 | 0, 4-12 | GP, BC |
| 9 | Kumar et al. (2018) | India | Asia | - | 1.0-21 | 0, 2.5-10 | GP |
| 10 | Ranjan et al. (2021) | India | Asia | Cobb | 1.0-42 | 0, 2.5-7.5 | GP |
| 11 | Rout et al. (2017) | India | Asia | Color synthetic | 1.0-21 | 0, 10-15 | GP |
| 12 | Samad et al. (2020) | Malaysia | Asia | - | 1.0-21 | 0, 5-15 | GP |
| 13 | Shambhvi et al. (2020) | India | Asia | Cobb | 1.0-42 | 0, 2.5 | GP, CT, OW |
| 14 | Sharma et al. (2020) | India | Asia | Cobb | 1.0-35 | 0, 1.5-5.5 | GP |
| 15 | Yassar et al. (2025) | Nigeria | Africa | Cobb | 1.0-42 | 0, 10-30 | GP |
| 16 | AL-Rekab et al. (2020) | Iraq | Asia | Ross | 1.0-42 | 0, 5-20 | BC |
| 17 | Ara et al. (2015) | India | Asia | Cobb | 7.0-42 | 0, 5-20 | GP, CT |
| 18 | Basak et al. (2002) | Bangladesh | Asia | Cobb | 7.0-42 | 0, 5-15 | GP, CT |
| 19 | Chichilichi et al. (2015) | India | Asia | - | 1.0-42 | 0, 5-10 | OW |
| 20 | Hassen et al. (2019) | Ethiopia | Africa | Cobb | 1.0-42 | 0, 2.5-7.5 | GP, CT, OW |
| 21 | Ibrahim et al. (2024) | Egypt | Africa | Cobb | 1.0-42 | 0, 4-12 | GP |
| 22 | Keser et al. (2024) | Turkey | Europe | Cobb | 1.0-21 | 0, 3-5 | GP |
| 23 | Khan et al. (2024) | Pakistan | Asia | Cobb | 1.0-35 | 0, 0.1-0.3 | GP |
| 24 | Kumar et al. (2018b) | India | Asia | - | 1.0-42 | 0, 2.5-10 | OW |
| 25 | Naghshi et al. (2014) | Iran | Asia | Cobb | 1.0-42 | 0, 5-15 | GP, BC, OW |
| 26 | Najim et al. (2022) | Iraq | Asia | Ross | 1.0-21 | 0, 5-30 | GP, BC |
| 27 | Paudel et al. (2015) | Nepal | Asia | Hubbard | 1.0-42 | 0, 10-20 | GP |
| 28 | Rengma et al. (2019) | India | Asia | Cobb | 1.0-42 | 0, 5-15 | GP, BC, OW |

*PP* production phase; *GP* growth performance; *BC* blood chemistry; *OW* organ weight; *CT* carcass traits.

| 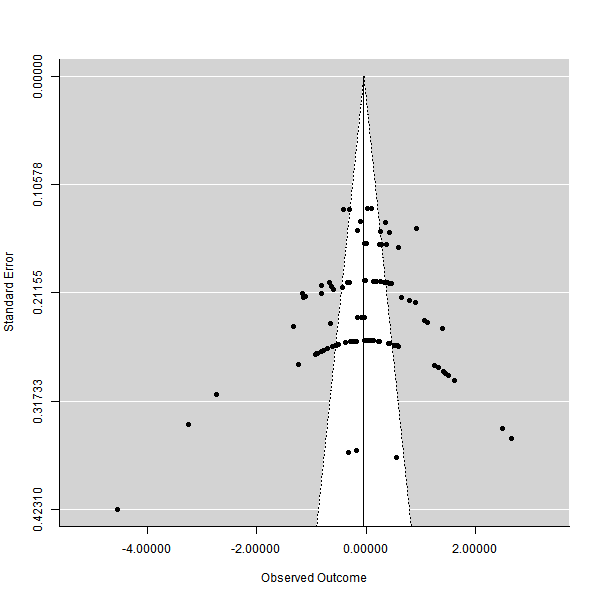**a** | | 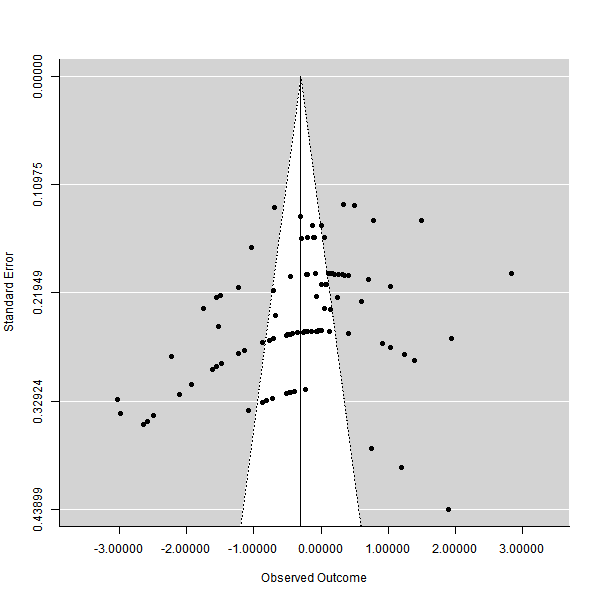  **b** | |  |
| --- | --- | --- | --- | --- |
| 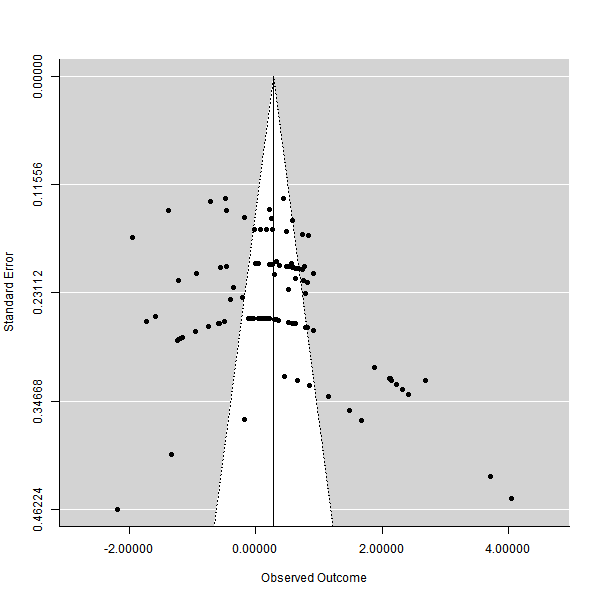  **c** | |  | |  |

**Supplementary Figure S1**. Forest plots of the effect of Azolla pinnata on (a) feed intake, (b) FCR, and (c) ADG of broilers

**References**

Abd El-Kareem M, Mousa MA, Mosaad GM (2025) The impact of feeding sun-dried azolla meal on growth performance and some serum biochemical parameters of broiler chickens. International Journal of Comprehensive Veterinary Research, **3**, 1-10.

Abdelatty AM, Mandouh MI, Al-Mokaddem AK, Mansour HA, Khalil HMA, Elolimy AA. Bionaz M. (2020) Influence of level of inclusion of azolla leaf meal on growth performance, meat quality and skeletal muscle p70S6 Kinase α abundance in broiler chickens. Animal, **14**, 2423–2432.

Abdelatty AM, Mandouh MI, Mohamed SA, Busato S, Badr OAM, Bionaz M, AlMokaddem AK, Moustafa MMA, Farid OAA, Al-Mokaddem AK. (2021) Azolla leaf meal at 5% of the diet improves growth performance, intestinal morphology and p70S6K1 Activation, and affects cecal microbiota in broiler chicken. Animal, **15**, 100362. doi:10.1016/j.animal.2021.100362.

AL-Hamed AM, Al-Husseiny NA (2023) Use of azolla plant in broiler diets and its effect on productive performance. IOP Conf. Series: Earth and Environmental Science, **1252**, 012134. doi:10.1088/1755-1315/1252/1/012134.

AL-Rekabi MM, Ali NA, Abbas FR (2020) Effect of partial and total substitution for azolla plant (*Azolla pinnata*) powder instead of soybean meal in broiler chicken diets on blood biochemical traits. Plant Archives, **20**, 1344-1348.

Ara S, Adil S, Banday MT, Khan MA (2015) Feeding potential of aquatic fern-azolla in broiler chicken ration. Journal of Poultry Science and Technology, **3**, 15-19.

Arram HM, Abdel Aal MH, Iraqi MM, El-Sayed AIM, Radwan AA (2023) Effect of azolla and probiotic feeding on broilers performance, and blood parameter traits. Egyptian Poultry Science, **43**, 333-347.

Basak B, Pramanik AH, Rahman MS, Tarafdar SU, Roy BC (2002) Azolla (*Azolla pinnata*) as a feed ingredient in broiler ration. International Journal of Poultry Science, **1**, 29-34.

Chichilichi B, Mohanty GP, Mishra SK, Pradhan CR, Behura NC, Das A, Behera K (2015) Effect of partial supplementation of sun-dried Azolla as a protein source on the immunity and antioxidant status of commercial broilers, Veterinary World, **8**, 1126-1130.

Dhumal MV, Siddiqui MF, Siddiqui MBA, Avari PE (2009) Performance of broilers fed on different levels of azolla meal. Indian Journal of Poultry Science, **44**, 65-68.

Hassen W, Tafese W, Amza N, Gudeta S, Beyene A, Muleta E (2019) Effect of partial substitution of soybean meal (*Glycine max*) by mosquito fern (*Azolla pinnata*) on growth performance and carcass characteristic of Cobb500 broiler chickens. Ethiopian Journal of Applied Science and Technology, **10**, 22-28.

Ibrahim S, Ateya A, Abdo M (2024) Economic evaluation of using azolla on growth performance of broiler chickens: gene expression impact. Egyptian Journal of Veterinary Science, **55**, 29-40.

Islam MA, Nishibori M (2017) Use of multivitamin, acidifier and Azolla in the diet of broiler chickens. Asian-Australasian Journal of Animal Science, **00**, 1-7.

Kamel ER, Hamed E (2021) Effect of dried azolla on growth performance, hematological, biochemical, antioxidant parameters, and economic efficiency of broiler chickens. Advances in Animal and Veterinary Sciences, **9**, 1886.

Keser O, Yigit F, Bilgin AS, Abas I, Bilal T, Kutay HC (2025) Effect of dietary Azolla supplementation on growth performance and histomorphometric features of the small intestine in broilers. Medycyna Weterynaryjna, **81**, 185-192.

Khan FU, Ullah R, Kinkpe L, Hassan SU, Ahamba IS, Goswami N, Binobead MA, Cedric AMA, Ahmed HQ, Shuaib M (2024) Substitution of soybean meal with *Azolla pinnata* meal improves gut histomorphology and growth performance in commercial broilers. Brazilian Journal of Poultry Science, **26**, 001-012.

Kumar M, Dhuria R, Jain D, Sharma T, Nehra R, Gupta L (2018b) Effect of Supplementation of Azolla on the hematology, immunity and gastrointestinal profile of broilers. International Journal of Livestock Research, **8**, 184-191.

Kumar M, Dhuria RK, Jain D, Sharma T, Nehra R (2018) Performance of broilers different phases fed on different levels of Azolla meal. Journal of Entomology and Zoology Studies, **6**, 792-795

Naghshi H, Khojasteh S, Jafari M (2014) Investigation the effect of different levels of Azolla (*Azolla pinnata*) on performance and carcass characteristics of Cobb broiler chicks. International Journal of Farming and Allied Science, **3**, 45-49.

Najim YS, Mohammed TT, Hussain FM (2022) The effect of the use of different levels of azolla to male broilers diets in the productive and physiological performance. Journal of Life Science and Applied Research, **3**, 43–48.

Paudel DR, Dhakal P, Timsina KP, Dahal A (2015) Azolla as an economic substitute to soybean-based feed for poultry. International Journal of Applied Science and Biotechnology, **3**, 619-625.

Ranjan VK, Sahu SP, Shekhar S, Kumar S, Singh SK, Kumari, R (2021) Influence of dietary supplementation of dried azolla on the growth performance of broiler chickens. International Journal Current Microbiology and Applied Science, **10**, 706-712.

Rengma DJ, Savino N, Vidyarthi VK (2019) Effect of dietary inclusion of azolla powder on performance of broiler chicken. Livestock Research, **07**, 144-150.

Rout SS, Pradhan CR, Mishra SK, Pati PK, Bagh J (2017). Performance of coloured synthetic broiler chicken fed dried azolla as protein substitute. International Journal Current Microbiology and Applied Science, **6**, 2349-2358.

Samad FAA, Idris LH, Hassim HA, Goh YM, Loh TC (2020) Effects of *Azolla spp*. as feed ingredient on the growth performance and nutrient digestibility of broiler chicken. Journal of Animal Physiology and Animal Nutrition, **00**, 1-8.

Shambhvi M, Katoch S, Chauhan P, Mane BG (2021). Effect of feeding *Azolla pinnata* in combination with direct-fed microbial on broiler performance. Tropical Animal Health and Production, **53**, 5. <https://doi.org/10.1007/s11250-020-02437-w>

Sharma RK, Pathak AK, Sharma RK, Sharma N (2020) Azolla cultivation to produce sustainable feed ingredient: chemical composition and its impact on performance of broiler chickens. Journal of Animal Research, **10**, 1067-1075.

Yassar FB, Rano NB, Abubakar A (2025) Nutritional evaluation of *Azolla pinnata* meal in broiler chicken diets. Agricultural Reviews, **4**, 1-6. doi: 10.18805/ag.RF-347.
